# A secondary analysis investigating therapist-effects in a guided internet-based intervention for adults with symptoms of depression

**DOI:** 10.1016/j.invent.2026.100964

**Published:** 2026-06-16

**Authors:** Manuel Heinrich, Pavle Zagorscak, Lars Schulze, Christine Knaevelsrud

**Affiliations:** a Freie Universität Berlin, Department of Education and Psychology, Division of Clinical-Psychological Intervention, Berlin, Germany; b Deutsches Zentrum Psychische Gesundheit, Standort Berlin, Germany; c Freie Universität Berlin, Department of Education and Psychology, Division of Clinical Psychology and Psychotherapy, Germany

**Keywords:** Therapist-effects, Internet-based intervention (IBI), Internet-based CBT (iCBT), Depression, Secondary analysis, Randomized-controlled trial (RCT)

## Abstract

**Objective:**

Studies consistently show that guided internet-based interventions (IBIs) are more effective than unguided IBIs for depression. Yet, little is known about whether average symptom change and rates of treatment discontinuation differ between therapists who guide clients through IBIs (IBI therapists).

**Method:**

This secondary analysis draws on data from three randomized controlled trials (RCTs; RCT1: *n* = 543, number of therapists *k* = 40; RCT2: *n* = 2277, *k* = 21; RCT3: *n* = 1769, *k* = 10) that provided adults with depression access to the same IBI. IBI therapists provided written, module-wise feedback and were available for on-demand contact. We examined therapist effects in (i) treatment discontinuation rates, (ii) rates of meaningful symptom improvements (≥50% improvement), and (iii) symptom change. In addition, we tested the association of these outcomes with the number of previously treated clients and the length of feedback messages.

**Results:**

Estimated therapist effects for treatment discontinuation were cVPC = 0.3 to 7.5% and cMOR = 1.081 to 1.617; for treatment response, cVPC = 0.4 to 3.1% and cMOR = 1.088 to 1.332; and for symptom change, conditional ICC = 0.2 to 0.7% and conditional median absolute difference = 0.121 to 0.275 PHQ-9 points. No association between the number of treated clients and any outcome emerged. However, longer feedback was associated with a reduced likelihood of treatment discontinuation.

**Conclusion:**

The between-therapist differences in discontinuation and symptom change in guided IBIs for depression can be small.

## Highlights

• We evaluated therapist effects in discontinuation, response, and change in a guided IBIs for depression across three RCTs.
• Results suggest that therapist effects were negligible to small.
• The number of previously treated clients did not consistently predict outcomes.

## 1 Introduction

Internet-based interventions (IBIs) are effective treatments for individuals with *depression* (e.g., Karyotaki et al., 2021; Moshe et al., 2021). Guidance (i.e., *contact and feedback*) provided by IBI therapists (i.e., individuals who guide clients through the program, with or without formal qualification as a psychotherapist) is an important treatment ingredient associated with higher symptom improvements and lowered rates of treatment discontinuation compared to unguided IBIs (Karyotaki et al., 2021; Krieger et al., 2023; Moshe et al., 2021; Musiat et al., 2022). Although guidance is beneficial on average, its quality within the same IBI can vary depending on the IBI therapist. These differences potentially lead to *therapist effects,* i.e., symptom improvements and discontinuation rates that vary between IBI therapists.

IBI therapists guide clients through pre-structured interventions tailored to the needs of an *average* client from a specific target population (e.g., individuals with mild to moderate depressive symptoms). They provide feedback, explain the intervention, reinforce commitment to the program, and provide support in case of symptom deterioration and technical difficulties. Although IBIs are highly structured, they still allow for individual therapist behaviors, as various qualitative studies indicate. For example, Holländare et al. (2016) analyzed written feedback messages in an IBI for depression and identified eight distinct categories of therapist behaviors. The most common of which were encouragement and affirmation (e.g., validation, normalization, and summarizing; Holländare et al., 2016). Moreover, IBI-therapists may differ in the extent to which they tailor feedback to clients' needs and/or provide feedback with varying levels of immediacy (see González-Robles et al., 2024, for a review of therapist behaviors in digital interventions). These differences could affect how supported participants feel, potentially influencing their engagement with the treatment modules and the benefits they derive from them. Consequently, these variations in IBI therapist behaviors may contribute to therapist effects.

At the same time, the role of IBI therapists differs from that in face-to-face (f2f) interventions. f2f-interventions consist of a blend of effects related to the methods chosen by a therapist and effects related to the therapist's behavior. As therapists typically select and deliver psychotherapeutic methods depending on their subjective assessment of clients' needs, the effects of the psychotherapeutic method and the therapist are often intertwined, especially in routine care settings. Therapist behavior and therapeutic approach form a single, undifferentiable treatment package. In contrast, IBIs are typically standardized interventions (i.e., all individuals have access to the same intervention, or they decide for themselves which of the largely standardized treatment modules they use). Therefore, therapists' effects in IBI are more distinguishable than in f2f-interventions. As such, it is possible to decompose the effect of an IBI into the effect of providing individuals with access to the treatment material and the contributions of the IBI therapist.

Numerous studies have examined therapist effects in f2f-psychotherapy. Although there is considerable between-study heterogeneity, maybe caused by the context of the study, inclusion criteria, and kind of intervention, many studies indicate that therapist factors often account for up to 15% of the variance in symptom improvement and discontinuation rates (Baldwin and Imel, 2013; Deisenhofer et al., 2025; Firth et al., 2019; Kim et al., 2006; Mehta et al., 2019; Nissen-Lie et al., 2024; Saxon et al., 2017; Sayer et al., 2022; Wampold and Brown, 2005; Willis et al., 2021; Xiao et al., 2023; Zimmermann et al., 2017).

However, to the best of our knowledge, only one study considered therapist effects in a guided IBI for depression similar to ours. This might be due to assumptions that IBIs leave limited room for therapist effects, given that they are highly manualized and structured. Almlöv et al. (2009) investigated heterogeneity among 10 IBI therapists (i.e., psychology students who had received specific clinical training) in treatment outcomes in a guided IBI for depression, each of whom provided asynchronous written feedback to approximately 10 clients. Therapist effects accounted for 7% to 11% of the variance in depression (i.e., BDI and MADRS-S), 11% in quality of life, but 0% in anxiety. Although the estimates were not statistically significant, the point estimates are comparable in size to estimates derived in f2f-psychotherapy. The discrepancy between theoretical expectation and these point estimates motivated a further investigation of therapist heterogeneity.

While the study conducted by Almlöv et al. (2009) provides valuable insights, it is subject to several limitations. *First*, the study relied on small sample sizes and included only a limited number of IBI therapists treating a limited number of clients, thereby reducing the generalizability of its findings. *Second*, the study focused solely on symptom outcomes at post-assessment, neglecting important variables in which therapist effects may become evident (e.g., treatment discontinuation). *Third*, due to the small sample size, the study could not investigate whether IBI therapist-related factors – such as the number of clients treated or the length of the feedback messages – are associated with treatment outcomes.

While prior research in f2f-psychotherapy found the effects of the number of treated clients on the average treatment outcome to be negligible to small (Germer et al., 2022; Goldberg et al., 2016), no study has investigated this association in IBIs. Studies on individualization have mostly focused on the qualitative features of IBI therapists' feedback messages (e.g., González-Robles et al., 2024), rather than the association between the feedback length and treatment outcome. Feedback length may serve as a useful proxy for individualization in the context of IBIs, as longer messages may reflect greater therapist engagement, more tailored responses to patient progress, and an increased effort to address individual needs within standardized treatment frameworks. Additionally, feedback length can also be readily monitored within digital treatment platforms, making it possible to detect deviations from a therapist's usual level of written feedback.

The present analysis aims to address these gaps described above by drawing on data from three randomized controlled trials (RCTs). Specifically, we explore therapist effects in rates of discontinuation, rates of meaningful symptom improvement (≥ 50% PHQ-9 reduction), and average symptom improvement measured with the PHQ-9 while on treatment. For this, we analyze a large sample of self-selected adults with depression who participated in a standardized CBT-based IBI, in which IBI therapists provided written feedback on each module and contact on demand. *Furthermore*, we explored whether the number of treated clients and the length of feedback are associated with the outcomes.

## 2 Method

This section reports how we determined our sample size, all data exclusions, all manipulations, and all measures in the study. This study was not pre-registered. It is an exploratory secondary analysis of trial data.

### 2.1 Samples

We use data from three RCTs providing self-selected adults with depression access to a CBT-based IBI for depression (Brose et al., 2023; Heinrich et al., 2025; Zagorscak et al., 2018). Due to differences in inclusion criteria, the qualification of IBI therapists, and the number of clients per IBI therapist across the trials, we analyzed the datasets separately rather than pooling them. The Ethics Committee of the Freie Universität Berlin, Department of Education and Psychology, approved all three studies (Approval numbers: RCT1: 70/2013, RCT2: 125/2016, RCT3: 004/2020). All trials were prospectively registered: RCT1: Australian New Zealand Clinical Trials Registry, ACTRN12614000312640; RCT2: Australian New Zealand Clinical Trials Registry, ACTRN12616001521415; RCT3: German Clinical Trials Register, DRKS00021106).

The trials applied the following depression-related inclusion criteria assessed at baseline: RCT1 and RCT2: 14 ≤ BDI-II ≤ 28; RCT3: 14 ≤ BDI-II, PHQ-9 ≤ 20. No formal diagnosis of depression was required. All trials excluded individuals with elevated levels of suicidality (RCT1 and RCT2: BDI-II item 9 score >1; RCT3: BDI-II item 9 score or PHQ-9 item 9 score >1) and excluded individuals with current or lifetime hypo(manic) episodes or psychosis as assessed using a telephone-based interview. Comorbidities (e.g., anxiety disorders) were not assessed systematically. There were no restrictions on which treatments individuals could initiate in parallel after randomization.

We excluded participants who did not begin working with the IBI after randomization (i.e., they did not enter the first treatment module; reason 1 [R1]) from the analysis. These individuals had *no* contact with an IBI therapist. Consequently, their non-initiation is unrelated to therapist behavior, and their exclusion is unlikely to bias the estimates of therapist effects. However, this shifts the interpretation of our findings to the subgroup of participants who would engage with the assigned treatment regardless of which IBI therapist they were allocated to (Kahan et al., 2023). Moreover, we excluded individuals who were assigned to another IBI therapist during the treatment (e.g., when the IBI therapist could not complete the treatments due to health-related reasons; R2). This criterion was relevant only to RCT3.

RCT1 was conducted between 2013 and 2015 and compared individualized versus standardized feedback (Zagorscak et al., 2018). Participants assigned to the standardized feedback arm were excluded from the analysis because they did not receive individualized feedback from an IBI therapist. From the individualized feedback arm, we included 543 of 555 randomized individuals (97.8%; 12 excluded due to R1).

RCT2 was conducted between 2016 and 2020 and investigated the effects of the sequencing of treatment content (Brose et al., 2023). Participants in the behavioral-activation-first (BAF) arm received access to the behavioral activation modules before the cognitive restructuring modules. Individuals in the cognitive-restructuring-first (CRF) arm had the sequence reversed. Both treatment arms were included. We analyzed 2277 of 2304 (98.8%; excluded due to R1: *n* = 27) randomized individuals.

RCT3 was conducted between 2020 and 2022 and examined the efficacy of IBI compared with a waitlist/unstandardized care-as-usual condition (Heinrich et al., 2025). Participants in arm 1 received immediate access to the IBI, while those in arm 2 gained access after an eight-week delay. Both treatment arms were included in the analysis. We analyzed 1769 of 1899 (93.2%; excluded due to R1: *n* = 110; R2: *n* = 20) randomized individuals. The number of persons not included is slightly higher in RCT3 because some individuals did not return after the waiting period (90 of 110).

Taken together, the sample under investigation is best described as a heterogeneous self-selected sample of adults interested in using the IBI to handle their self-reported symptoms of depression who at least started to work with the first treatment module once they were randomized. Table 1 summarizes major sample characteristics of all three samples. Given that this is an exploratory secondary analysis of available trial data, no power analysis was conducted for the research question under investigation in this manuscript.

**Table 1:** Sample characteristics.

Variable	RCT1	RCT2	RCT3
**Study Characteristics**			
cases from initial trial, n (%)	543 (97.8)	2277 (98.8)	1769 (93.2)
PAF, *n* (%)	543 (100.0)	1099 (48.3)	950 (53.7)
CRF, *n* (%)	0 (0.0)	1178 (51.7)	0 (0.0)
CAU, *n* (%)	0 (0.0)	0 (0.0)	819 (46.3)
**Therapist Characteristics**			
Number of Therapists	40	21	10
Number of Clients/Therapist, Mdn (min, max)	13.5 (2, 33)	59.0 (4, 297)	191.0 (10, 288)
Number of Responders/Therapist, Mdn (Min, Max)	2.0 (0, 6)	14.0 (1, 71)	53.0 (0, 76)
Number of Discontinuers/Therapist, Mdn (Min, Max)	4.5 (0, 14)	20.0 (2, 91)	55.0 (5, 86)
Symptom Change/Therapist, Mdn (Min, Max)	−4.0 (−1.4, −7.0)	−3.4 (−2.6, −5.2)	−3.1 (−2.5, −4.1)
**Client Characteristics**			
Age, M (SD)	44.9 (11.6)	42.3 (12.9)	39.0 (11.5)
Female, *n* (%)	353 (65.0)	1356 (59.6)	1162 (65.7)
Education			
No certificate, *n* (%)	4 (0.7)	2 (0.1)	1 (0.1)
Lower secondary, *n* (%)	34 (6.3)	85 (3.7)	33 (1.9)
Secondary school, *n* (%)	118 (21.7)	378 (16.6)	220 (12.4)
Trade school, *n* (%)	117 (21.5)	564 (24.8)	419 (23.7)
College, *n* (%)	82 (15.1)	256 (11.2)	202 (11.4)
University, *n* (%)	188 (34.6)	992 (43.6)	894 (50.5)
BDI-II baseline, *M* (*SD*)	22.0 (3.9)	21.5 (4.1)	25.2 (6.6)
PHQ-9 baseline, *M* (*SD*)	11.9 (3.4)	11.7 (3.3)	12.9 (3.5)
current MDE, *n* (%)	269 (49.5)	710 (31.2)	872 (49.3)

*Note*. BDI-II = Beck Depression Inventory-II; CAU = Care-as-Usual; CRF = Cognitive Restructuring First; GAD-7 = Generalized Anxiety Disorder 7; MDE = Major Depressive Episode, Current MDE was assessed in a telephone-based SCID interview before participation; PAF = Positive Activation First; PHQ-9 = Patient Health Questionnaire-9.

### 2.2 Intervention

All RCTs relied on the same guided CBT-based IBI that includes two modules on structured writing, two modules each on behavioral activation and cognitive restructuring, and a final task on expressive writing. The module order was the same in all treatment arms, except for the CRF arm in RCT2. Detailed descriptions of the individual modules are provided elsewhere (Heinrich et al., 2025).

All modules follow the same structure: (1) participants review therapist feedback on their previous work, (2) receive multimedia-based psychoeducation, and (3) are given instructions and tools for completing an upcoming therapeutic task (e.g., structured writing, entries to daily planners, or thought protocols). Participants completed models sequentially; that is, they could not simply jump to the next module; instead, they had to wait until the IBI therapist had provided feedback on their work with the modules.

### 2.3 IBI therapists and written feedback

The number of IBI therapists was 40 (RCT1), 21 (RCT2), and 10 (RCT3). The median number of clients treated per IBI therapist was lowest in RCT1 (*Mdn =* 14) and considerably larger in RCT2 (*Mdn =* 59) and RCT3 (*Mdn =* 191). See Table 1 for further details. The IBI therapists varied in their qualifications. In RCT1, the IBI therapists held a Master's or Bachelor's degree in psychology but no qualification as f2f psychotherapists. In RCT2 and 3, they were either fully licensed f2f-psychotherapists or had a provisional license as part of their advanced training in psychotherapy. In RCT2 and 3, IBI therapists guided clients through both treatment variants. All IBI therapists are self-selected and thus willing to provide IBI treatments for adults with depression. Except for RCT1, most IBI therapists were publicly listed on the university's website with photos, allowing clients to verify that real individuals were guiding them through the IBI.

IBI therapists used an internet-based platform that provided an overview of all assigned clients, their treatment progress, and module-specific symptom ratings (PHQ-9 scores). The platform functioned as a communication hub for delivering written feedback and, when necessary, additional messages.

The IBI therapists initiated contact with clients via a written welcome message. IBI therapists provide task-related written feedback after participants complete structured writing tasks (Modules 1, 2, and 7) or after they have had time to engage with the module content for seven days (Modules 3 to 6). Participants received automated email reminders at 4, 7, 14, and 20 days of inactivity (i.e., failure to log in). In addition, IBI therapists – or the clinical supervisor of RCT1 and RCT2 – offered supportive telephone contact in response to symptom deterioration or reported suicidality. These messages were based on semi-standardized text blocks pre-structured for each module, which IBI therapists individualized to reflect the client's specific situation and the extent and quality of their task completion. IBI therapists were required to provide feedback within 2 working days (Monday through Friday) after the participant had completed a module. If an IBI therapist missed this deadline, the supervisor was automatically notified via email. In addition to scheduled feedback, therapists and clients could exchange further messages through a “contact on demand” feature. IBI therapists reported that clients primarily used this option to address technical issues or scheduling concerns (e.g., vacation plans).

All IBI therapists received training on the IBI and its components before treating their first clients. In addition, IBI therapists received a detailed manual outlining how to deliver written guidance for each treatment module. The intervention manual is available on reasonable request. To ensure adherence to the treatment protocol, a supervisor reviewed and evaluated each IBI therapist's first two feedback messages for each module. This quality assurance process was implemented to promote fidelity to the intervention manual. A single person largely provided clinical supervision.

### 2.4 Questionnaire data

Demographic information, including age, sex, and level of education, was collected during baseline assessment. Depressive symptoms were measured using the Patient Health Questionnaire-9 (PHQ-9; range: 0–27), which was administered at baseline, post-assessment, and before Modules 1, 3, 4, 5, 6, and 7. No data was collected before Module 2, because Modules 1 and 2 were intended to be completed in a single week.

### 2.5 Criterion variables

The current study focuses on variables related to behavior and symptom change while on treatment.

Criterion (1) is *treatment discontinuation*, defined as not starting to work with treatment module 7 (i.e., the last treatment module). A low discontinuation rate may indicate that the IBI therapist's behavior encourages participants to engage with every treatment module, regardless of whether clients are dissatisfied with the program or feel they have achieved either sufficient or insufficient improvements. Thus, this outcome reflects a central task of an IBI therapist: maintaining participant engagement to increase the “dose” of intervention material received.

Criterion (2) evaluates the rate of *meaningful improvement*, defined as a ≥ 50% reduction in symptoms from the assessment collected before module 1 to the last available pre-module assessment. The ≥50% improvement cut-off is often used to operationalize treatment response (e.g., Cuijpers et al., 2021). Patients with only pre-module 1 assessments were coded as experiencing no change.

Criterion (3) considers *average symptom improvement* calculated by subtracting the score from the assessment collected before module 1 from the last available pre-module assessment (i.e., negative scores indicate symptom improvement).

Criteria (2) and (3) rely on the *while-on-treatment* concept, that is, measuring the amount of symptom change and meaningful symptom improvement up to starting the last treatment module a randomized client worked with (ICH, 2019). The estimand was therefore heterogeneity between therapists in the average symptom change and response while clients used the IBI, which implies that clients may have received varying doses of treatment. This outcome conceptualized the therapist's role as keeping participants engaged until they had either experienced symptom improvement or had completed all treatment modules. As these effects consider only outcomes before discontinuing or completing the IBI, they have no missing data.

As all individuals received the same standardized treatment material, variations in outcomes can be attributed to differences in how IBI therapists guided participants through the IBI, rather than differences in the intervention itself.

### 2.6 Statistical analysis

All research questions were addressed using Bayesian multilevel models. All models were estimated using *brms* with default priors (Bürkner, 2017). All models demonstrated satisfactory convergence, with *R*-hat values below 1.05. There were no divergent transitions. Results are reported as posterior means accompanied by equitail 95% credibility intervals (CRI) that cover the central 95% of the parameter's posterior distribution. Although not intended as such, CRIs that do not include zero indicate statistical significance.

### 2.7 Heterogeneity among IBI therapists

We applied Bayesian multilevel logistic regression models for Criterion 1 (*treatment discontinuation)* and Criterion 2 *(meaningful symptom improvement),* and Bayesian multilevel linear regression models for Criterion 3 (*PHQ-9 symptom change*). In all models, IBI therapists were included as random effects, reflecting the assumption that they are drawn from a common population. We provide all model equations in the online supplemental material. *R* scripts and outputs are provided in the corresponding OSF project: https://osf.io/h8uyw/overview?view_only=a594e1fd48a34452b5f09ad298eea2d0

Models were estimated separately for each study, as studies differed in inclusion criteria and were conducted across different years, potentially introducing cohort effects related to broader changes in internet use and acceptance of digital interventions.

Clients were assigned consecutively to IBI therapists. Since IBI therapists had different contracts, they treated varying numbers of clients. However, because the assignment was not based on specific covariates (e.g., more severely impaired patients were assigned to IBI therapist A), there should be *no* systematic confounding. However, to account for random variation among the clients of the IBI therapists in theoretically important covariates, all models included clients' sex (coded as 0 = female), age (centered at 40 years), and baseline depression severity (PHQ-9, centered at 10) as fixed-effect covariates. For participants assigned to the waitlist condition, we used the depression score assessed after the post-waitlist period as the baseline measure. For data from RCT2 and 3, we also added the study arm as a dummy-coded covariate. Thus, we assume that the therapist effects are the same in both treatment conditions. Plots showing the distribution of these covariates within each IBI therapist are shown in the Supplemental Material.

For logistic models, we describe heterogeneity among IBI therapists using the variance partition coefficient (VPC; Goldstein et al., 2002) and the median odds ratio (MOR; Larsen, 2005). The VPC ranges from 0 to 100%, with higher values indicating greater variability among therapists. The VPC is comparable to the intra-class correlation coefficient (ICC), with the within-cluster variance fixed at π^2^/3 (Goldstein et al., 2002). Ratios of variance, such as those provided by the VPC (and ICC), can be difficult to interpret. To allow for a more nuanced evaluation, we add the Median Odds-Ratio (MOR) as a further indicator.

The MOR ranges from 1 to infinity, with higher values reflecting more therapist heterogeneity (Larsen, 2005). The MOR focuses on comparing randomly selected therapists. A thought experiment illustrates the underlying idea. Consider sampling two therapists from a normally distributed random-effects distribution. If these therapists could treat the same patient in two parallel universes, then the only difference in the patient's outcome would arise from the difference between the two therapists. This difference can be described in terms of an odds ratio, with the therapist having the smaller random effect used as the reference (Larsen, 2005). Repeating this process (i.e., sampling two random effects and computing the odds ratio) yields a distribution of odds ratios. The MOR is the median of that distribution. Thus, a MOR of 1.5 indicates that, in 50% of comparisons between two randomly selected IBI therapists, the OR would be less than 1.5.

For linear models, we report the ICC (Raudenbush and Bryk, 2002) and the Median Absolute Difference (MAD). The ICC ranges from 0 to 100%, with larger values indicating greater between-therapist differences. The MAD exploits the assumption that the random effects are normally distributed and ranges from 0 to the scale's maximum. The MAD is similar to the MOR but focuses on differences in expected outcomes rather than odds ratios (see the Supplement for a derivation). Specifically, the MAD is the median of the absolute differences in the expected outcome, measured in units of the outcome measure (i.e., PHQ-9 points), when two therapists are randomly selected from the random-effects distribution and treat clients with identical covariate values. For example, a MAD of 0.5 indicates that in 50% of comparisons between two randomly selected therapists, the absolute difference in the expected outcome is less than 0.5 PHQ-9 points.

Because all models include covariates, all indices have a conditional interpretation. Thus, the conditional ICC (cICC), MAD (cMAD), MOR (cMOR), and VPC (cVPC) represent between-therapist variability among individuals with the same covariate values.

Various additional analyses were conducted. These include analyses of the pooled sample and of each treatment arm separately. Moreover, we conducted an analysis that included the number of treated clients and quarter-of-year dummies as covariates. Moreover, we conducted an analysis that included only individuals who began working with all treatment modules. Overall, the effects were highly comparable across the analyses. Therefore, we report the main analysis in the manuscript and refer interested readers to the supplemental Tables for details on the sensitivity analysis.

### 2.8 Predictors of outcomes

We explored the associations of the number of treated clients and the feedback length with the three outcome measures using Bayesian logistic or linear multilevel regression models. We again included baseline symptom severity, baseline age, and sex as fixed-effect covariates, with therapists modeled as random effects.

*First*, we examined the relationship between the outcome measures and the *number of clients a therapist has treated up to that client*. This analysis and the employed approach were motivated by similar research in f2f-psychotherapy (Germer et al., 2022; Goldberg et al., 2016). Within each IBI therapist, clients were ranked by the date the therapist sent the welcome message, with clients who received the message on the same day assigned the same rank. This variable was constructed only for RCT2 and RCT3, as the necessary data were unavailable for RCT1. We divided the rank by 10, so that a 1-unit increase corresponds to approximately 10 additional treated clients. Scaling by a factor of 10 increases the magnitude of the regression coefficient, which can aid interpretability when the coefficient is small in the original metric of the predictor. The rank number was included as a fixed effect in the model, assuming a linear relationship between the criterion and predictor.

*Second*, we examined the predictive value of the length of their feedback responses (and the deviation from the average length) to the expressive writing tasks in Modules 1 and 2 and the outcomes. The number of characters was divided by 500 (see above for interpretation). For each client, we first calculated the mean number of characters across the first two modules. Then we computed the therapist-specific mean and centered the clients' values relative to it (within-between decomposition; Bell et al., 2018). Thus, a positive deviation from the therapist's average indicates longer-than-usual feedback. Both the therapist's mean feedback length and each client's deviation from this mean were included as predictors in the model. These variables could be constructed for RCT1 and RCT3. For this analysis, we excluded individuals who did not complete the first two writing tasks (RCT1: 31; RCT3: 179). Comparisons of demographic (sex, age) and baseline clinical characteristics (PHQ score) between included and excluded individuals revealed no significant differences across the RCTs (all *p*s > 0.05). The only exception was RCT3, where the distribution of sex differed significantly between included and excluded individuals (χ^2^(1) = 4.72, *p* = 0.030), indicating that slightly more males discontinued within the first two treatment modules.

Given that the feedback length is a behavior in response to the client's behavior, the coefficients may *not* be interpreted *causally*. Instead, it reflects whether the IBI therapists' behavior in the first two modules can predict outcomes after those modules. Given that only individuals who completed the first two treatment modules are investigated, these estimates are not intended to provide an unbiased estimate of the association between feedback length and outcome among all randomized individuals, but only among those who complete the first two treatment modules.

## 3 Results

### 3.1 Heterogeneity among IBI therapists

Table 1 provides descriptive statistics summarizing each criterion and predictor variable. Table 2 summarizes the conditional indices (cICCs, cVPCs, cMADs, and cMORs) with their 95% credible intervals (CrIs). Supplemental tables present all parameter estimates, including the regression weights for the covariates.

**Table 2:** Heterogeneity between IBI therapists.

Criterion	Parameter	RCT1	RCT2	RCT3
		β [95% CRI]	β [95% CRI]	β [95% CRI]
Discontinuation	cVPC	0.075 [0.001, 0.209]	0.003 [0.000, 0.017]	0.004 [0.000, 0.021]
	cMOR	1.617 [1.045, 2.436]	1.081 [1.002, 1.257]	1.088 [1.003, 1.288]
Improvement	cVPC	0.031 [0.000, 0.108]	0.004 [0.000, 0.019]	0.004 [0.000, 0.021]
	cMOR	1.332 [1.017, 1.824]	1.095 [1.004, 1.276]	1.088 [1.003, 1.289]
Change	cICC	0.007 [0.000, 0.033]	0.005 [0.000, 0.017]	0.002 [0.000, 0.010]
	cMAD	0.275 [0.010, 0.753]	0.234 [0.018, 0.526]	0.121 [0.004, 0.393]

*Note.* cICC = conditional intra-class correlation. cMAD = conditional median absolute difference. cMOR = conditional marginal odds ratio. cVPC = conditional variance partition coefficient. [95% CRI] = 95% Credibility interval.

#### 3.1.1 Treatment discontinuation

Heterogeneity was largest in RCT1 (cVPC = 7.5%, cMOR = 1.617), but substantially smaller in RCT2 (cVPC = 0.3%, cMOR = 1.081) and RCT3 (cVPC = 0.4%, cMOR = 1.088). The estimates in RCT1 have the widest CRIs, indicating greater uncertainty.

#### 3.1.2 Meaningful symptom improvement

Heterogeneity was again largest in RCT1 (cVPC = 3.1%, cMOR = 1.332) and substantially smaller in RCT2 (cVPC = 0.4%, cMOR = 1.095) and RCT3 (cVPC = 0.4%, cMOR = 1.088). The estimates in RCT1 have the widest CRIs, indicating greater uncertainty.

#### 3.1.3 Average-symptom change

Heterogeneity was similar in all three RCTs; RCT1 (cICC = 0.7%, cMAD = 0.275), RCT2 (cICC = 0.5%, cMAD = 0.234), and RCT3 (cICC = 0.2%, cMAD = 0.121). The width of the CrIs was comparable across all three samples.

### 3.2 Predictors of outcomes

#### 3.2.1 Number of treated clients

Table 3 summarizes the estimated associations of the number of treated clients with treatment discontinuation, meaningful improvement, and symptom change. Note that RCT1 was not included in this analysis because the dataset lacked a variable indicating the exact date participants received their welcome message from their therapist, which was necessary to construct the rank order of clients treated. All parameter estimates are provided in the online supplemental material. Overall, there was no evidence for a relationship between the rank number of clients treated and discontinuation or meaningful improvement. Only in RCT2 did a small negative association emerge, indicating that expected average improvements decreased as the number of clients a therapist treated increased, *β* = 0.058, 95% CI [0.013, 0.103].

**Table 3:** Association of treatment discontinuation, meaningful improvement, and change with the number of treated clients.

Criterion	RCT2		RCT3	
	β [95% CRI]	OR [95% CRI]	β [95% CRI]	OR [95% CRI]
Dropout	0.010 [−0.016, 0.035]	1.010 [0.984, 1.036]	0.023 [−0.004, 0.049]	1.023 [0.996, 1.051]
Response	−0.015 [−0.039, 0.007]	0.985 [0.962, 1.007]	−0.000 [−0.025, 0.025]	1.000 [0.975, 1.025]
Change	0.058 [0.013, 0.103]	–	−0.005 [−0.052, 0.043]	–

*Note.* All parameters are estimated conditional on the covariates: age, sex, and baseline symptom severity, which were included as fixed effects in the multilevel models. β = Median of the parameters' posterior distribution. OR = Odds ratio. [95% CRI] = 95% Credibility interval.

#### 3.2.2 Feedback-length

There was considerable heterogeneity in the tendency to individualize feedback messages, with ICCs of 48.2% (RCT1) and 61.1% (RCT3). Table 4 presents the results for predicting the criterion variables using both within-therapist deviations (i.e., deviations from the therapist's mean level) and therapists' mean levels of feedback length (between-level) as predictors. Meaningful associations emerged only for treatment discontinuation. In RCT3, at the within-therapist level, clients were less likely to discontinue treatment when therapists wrote longer feedback messages. At the between-therapist level, longer feedback messages were associated with lower discontinuation rates in RCT1.

**Table 4:** The association of treatment discontinuation, response, and change with feedback length.

Level	Criterion	RCT1		RCT3	
		β [95% CRI]	OR [95% CRI]	β [95% CRI]	OR [95% CRI]
W	Dropout	−0.074 [−0.487, 0.319]	0.948 [0.614, 1.376]	−0.258 [−0.418, −0.101]	0.775 [0.658, 0.904]
W	Response	−0.010 [−0.216, 0.196]	0.995 [0.805, 1.216]	0.070 [−0.055, 0.195]	1.075 [0.946, 1.215]
W	Change	−0.099 [−0.538, 0.336]		−0.213 [−0.456, 0.026]	
B	Dropout	−0.879 [−1.358, −0.431]	0.427 [0.257, 0.650]	−0.024 [−0.239, 0.171]	0.982 [0.787, 1.186]
B	Response	0.092 [−0.146, 0.336]	1.105 [0.864, 1.399]	−0.035 [−0.184, 0.103]	0.968 [0.832, 1.108]
B	Change	−0.084 [−0.518, 0.347]		0.027 [−0.240, 0.287]	

*Note.* All parameters are estimated conditional on the covariates: age, sex, and baseline symptom severity, which were included as fixed effects in the multilevel models. One unit of feedback length equals 500 letters. Parameters with credibility intervals not including zero (or one if the parameter is an Odds-Ratio) are printed in bold. β = Median of the parameters' posterior distribution. OR = Odds ratio. [95% CRI] = 95% Credibility interval. W = within-therapist-centered feedback length. B = average amount of feedback length per therapist at the between-level.

## 4 Discussion

This exploratory secondary analysis examines therapist effects on treatment discontinuation rates, meaningful improvement, and average symptom improvements, using data from three RCTs providing adults with self-reported depressive symptoms access to a guided CBT-based IBI. Overall, therapist effects were small. There was no convincing evidence that the number of treated clients was associated with any of the three criteria. However, evidence suggests that longer feedback messages (i.e., more written text) are associated with lower odds of treatment discontinuation.

### 4.1 Heterogeneity between therapists

A consistent pattern emerged. Both the cVPCs and cICC were highest in RCT1 (cVPC: 7.5% for treatment discontinuation and 3.1% for meaningful improvement; cICC: 0.7% for symptom change) and substantially lower in RCT2 (cVPC: 0.3% and 0.4%; cICC: 0.5%) and RCT3 (cVPC: 0.4% and 0.4%; cICC: 0.2%). The width of the 95% CIs varied by outcome type and trial. For symptom change, the 95% CRIs covered similar ranges in all three RCTs (RCT1: 0–3%; RCT2: 0–1.7%; RCT3: 0–1.0%); all lie entirely below between-therapist variance typically reported in f2f-psychotherapy (Baldwin and Imel, 2013). In contrast, for binary outcomes – meaningful improvement and treatment discontinuation – the 95% CrI were considerably wider in RCT1 (discontinuation: 0–21%; meaningful improvement: 0–11%) compared to RCT2 and 3 (both outcomes: 0–2%). We attribute the larger posterior means and the wider 95% CrIs for the binary outcomes in RCT1 to an interaction between outcome type, the number of therapists, and the number of clients per therapist. Binary outcomes contain less information than continuous outcomes. The number of responders and discontinuers were much lower in RCT1 than in RCT2 and 3 (see Table 1). As a result, the low numbers in RCT1 likely led to imprecise estimates of within-therapist effects, which, in turn, contributed to the larger, less precise (and probably overestimated) estimates of the between-therapist variance in RCT1. The observation that these differences were smaller for the continuous outcome supports this reasoning. Thus, we consider the estimates from RCT2 and 3 to be more reliable than those from RCT1.

Conclusively, the differences between therapists in our IBI are likely negligible. From a clinical perspective, the low heterogeneity between therapists is promising. Previous studies underline that the inclusion of therapists or other forms of human contact is important for efficacy and especially for adherence (Karyotaki et al., 2021; Moshe et al., 2021). Our study suggests that it is possible to design IBIs so that it is less important which of the trained IBI therapists provides this guidance; our IBI therapists appear largely interchangeable in our trials. This interchangeability makes it much easier to communicate the expected benefit of a guided IBI to participants, as it is not necessary to differentiate among IBI therapists. In contrast, such statements are more difficult to make in the context of f2f-psychotherapy, where therapist effects are often estimated to be as large as 15% and sometimes considerably larger (Baldwin and Imel, 2013; Nissen-Lie et al., 2024).

In this study, all coaches received comparable training, were supervised, and followed established routines. Therefore, it is important to avoid overgeneralizing our findings. Our results do not suggest that therapist effects in IBI are generally absent or low. Their sizes can depend (a) on the IBI, (b) on the outcome, and (c) on the inclusion criteria. In addition, the IBI therapists in this study are not independent since they work at the same university. Therefore, therapist effects could be larger in real-world settings where IBI therapists are more independent from each other and do not share the same quality assurance processes. Therefore, future IBI studies should routinely report estimates of between-therapist heterogeneity in IBIs to generate a more comprehensive evidence base. However, despite this limitation, our results clearly suggest that it is possible to create a setting in which therapist effects are low.

Strictly speaking, we cannot rule out the possibility that the greater heterogeneity in rates of discontinuation and response rates observed in RCT1 is not just a method-related artifact but reflects real differences. Practice effects may have played a role – the differences between therapists may be more pronounced when they treat their first clients and few clients overall (as in RCT1) and decrease over time as they become more familiar with the treatment method through treating more clients (as was the case in RCT2 and 3). However, this explanation seems less likely, as there is no significant heterogeneity among IBI therapists in RCT1 regarding symptom change.

In addition, the formal qualifications of the IBI therapists may also have influenced the results. In RCT1, the IBI therapists were master's students of psychology and individuals already holding a master's degree in psychology. In contrast, the IBI therapists in RCT2 and RCT3 were either fully licensed psychotherapists or held provisional licenses as part of their advanced psychotherapy training. The qualification level was therefore inseparable from the respective trial, and any associated effects cannot be disentangled in the current design. If qualification effects on average outcomes exist, they are likely small (Baumeister et al., 2014; Moshe et al., 2021). However, even when groups with different qualifications produce similar average outcomes, one group may still show considerably more heterogeneity than the other (i.e., different therapist effects). Future studies should therefore include IBI therapists with different qualification levels within the same study, assign clients to therapists randomly, and explore whether qualification levels moderate levels of between-therapist heterogeneity.

### 4.2 Number of treated clients

The results indicate a close-to-zero association between the number of treated clients and treatment discontinuation and meaningful improvement. These findings indicate that neither the odds of treatment discontinuation nor the odds of meaningful improvement change with the number of treated clients. However, our study yielded some inconclusive results regarding the association between the number of previously treated clients and symptom improvements.

In RCT3, the model showed that for every additional 100 clients treated, the expected symptom improvement decreased by −0.050, 95% CI [−0.520, 0.430], PHQ-9 points, which is very close to zero. These close-to-zero findings are comparable to those from f2f-psychotherapy: Germer et al. (2022) analyzed data from 241 f2f-therapists treating approximately 3400 clients and found a close-to-zero decrease in the expected change of 0.03 standardized units per 100 clients treated. However, in RCT2, the expected mean improvements decreased slightly with an increasing number of clients a f2f-therapist had treated. Specifically, the model showed that for every additional 100 clients treated, the expected symptom improvement decreased by 0.58 PHQ-9 points, with a 95% CI of [0.130, 1.030]. This finding is similar to that of Goldberg et al. (2016), who found that outcomes decline with experience. In a study of 170 f2f-therapists and 6591 clients, Goldberg et al. (2016) reported that for each additional 100 clients treated, there was a decrease in the standardized expected outcome of 0.20 units. Given these inconsistencies, further research is needed to assess the impact of previously treated clients in this setting. This appears important, as IBI therapists can treat more clients in much less time.

### 4.3 Length of feedback messages

Overall, there was considerable heterogeneity in the average length of feedback messages between IBI therapists. Thus, IBI therapists tend to use the feedback templates differently. We found that longer feedback messages were associated with a lower likelihood of treatment discontinuation. In RCT3, this association was observed only at the within-therapist level. In contrast, in RCT1, the association emerged only at the between-therapists level. Thus, there is evidence for an association between the length of feedback messages and treatment discontinuation. We assume that the RCT-specific constellation of the number of IBI therapists (many in RCT1 and few in RCT3) and the number of treated clients per IBI therapist (few in RCT1 and many in RCT3) explains why a significant portion of the association between feedback length and treatment discontinuation shifts from within-level (RCT3) to between-level (RCT1) effects. It is probably a larger number of clients per therapist that allows for relevant within-level variation.

It is essential to note that deviations in feedback message length from the IBI-therapist's average likely reflect responses to client behavior in the preceding week, particularly in relation to what clients wrote in their expressive writing tasks. As such, we *cannot* infer that longer feedback messages *cause* lower rates of treatment discontinuation. Rather, message length may serve as a proxy for certain client characteristics that are themselves associated with treatment discontinuation. For example, highly motivated clients who are more open to the IBI might submit different materials than those with lower motivation, thereby providing therapists with more or fewer opportunities to respond. Thus, our findings should not be interpreted as evidence of a causal relationship between feedback length and treatment discontinuation. Nevertheless, one may use that deviation to inform therapists when providing treatments. For example, once the average behavior is established, the IBI program could automatically flag clients whose therapist responses differ from their typical responses (e.g., unusually short answers). Importantly, such deviation should not be interpreted as evidence of the therapist's inattentiveness. It may reflect client disengagement, therapist response behavior, or an interaction between the two. Rather, it should be treated as an indicator that the client is at a higher risk of discontinuing the program, irrespective of the underlying reason. Such an indicator could be combined with analyses of client messages sent to their therapist, as associations between the content of such messages and outcome have been found (Svartvatten et al., 2015). In response to such a flag, for example, a supervisor or the therapist could initiate a brief check-in call to discuss the client's experience with the treatment so far and bolster treatment expectations.

Moreover, we only considered the quantity, not the quality, of the feedback messages. However, González-Robles et al. (2024) showed that therapists can differ along various dimensions. In other words, the feedback length is likely a poor proxy, combining relevant (e.g., using encouragement when needed) with irrelevant and potentially harmful (e.g., ignoring the client's complaints) therapist behaviors related to feedback. Further studies should especially consider the interaction between the lengths of feedback and the qualitative features of therapist behaviors identified in studies on digital interventions (González-Robles et al., 2024).

## 5 Limitations and future directions

Our study addresses several common limitations of previous research on therapist effects in IBI, including small sample sizes and low numbers of clients per therapist. At the same time, it shares some methodological limitations with studies of therapist effects in psychotherapy more generally.

*First*, our study is a secondary analysis that was not specifically designed to examine the effects of therapists. Therefore, it was not powered to estimate therapist effects. Moreover, clients were assigned to IBI therapists in a sequential rather than a random manner. However, (1) the highly structured treatment setting, (2) the non-selective assignment of participants to therapists (i.e., we did not consider client characteristics when assigning them to therapists), and (3) the statistical adjustment for theoretically important covariates may have resulted in estimates close to those of a randomized design. Nonetheless, future research should randomly assign clients to therapists to strengthen causal inferences.

*Second*, we did not experimentally manipulate the therapists' behavior but rather investigated naturally occurring differences. Consequently, it is impossible to attribute the observed effects to a specific behavior. Future research could address this limitation by using factorial randomized controlled trials that systematically vary dimensions of therapist behavior, such as the timing of the response (immediate vs. delayed), the length of the message (brief vs. detailed), or the communication style (matter-of-fact vs. empathic). Such a study would allow for a clearer understanding of the causal effects of therapist behaviors.

*Third*, although the overall sample size was substantial, studies with larger numbers of clusters are needed, as larger clusters allow for more general conclusions. The related shortcomings in RCT1 (low number of clients per therapist) and RCT3 (low number of therapists) are attributable to the nature of our study as an exploratory secondary analysis, in which sample sizes are calculated to achieve a different goal. Given the scalability of IBIs, future studies that plan to investigate therapist effects should include larger samples, with the same number of clients randomly assigned to a set of trained therapists. Such studies will be well-positioned to provide more robust conclusions about therapist effects.

*Fourth*, we focused on depression only. Therapist effects may differ across different outcomes depending on the specific therapist behavior. Future studies should focus on other outcome measures, including anxiety, self-efficacy, and perceived social support. Future research may also consider therapists' behaviors as outcomes, such as their tendency to provide contact on demand or the amount of time spent writing feedback messages. Moreover, in this study, we focused on “while-on-treatment” effects, but long-term outcomes should be examined as well to explore whether therapist effects evolve.

## 6 Conclusion

Therapist effects in the IBI under investigation were negligibly small, suggesting that IBIs can be designed such that, on average, effectiveness and risk of dropout are not systematically associated with the IBI therapist who guided the client through the program. Moreover, the positive association between the feedback length and the therapist's specific reference level of feedback messages may serve as an indicator for clients at greater risk of dropping out.

## Declaration of Generative AI and AI-assisted technologies in the writing process

During the preparation of this work, the authors used ChatGPT, DeepL, and Grammarly to improve the quality and conciseness of the language. After using this tool/service, the author(s) reviewed and edited the content as needed and take(s) full responsibility for the content of the published article.

## Funding

In all three RCTs “Techniker Krankenkasse”, a German public health insurance company, paid for the treatment of participating individuals on a case-by-case basis. In RCT 1, additional funding was received for planning, execution, and analysis. At no point was the funding body involved in the study design, data collection, analysis, or interpretation, the writing of the report, or the decision to submit the article for publication.

## Declaration of competing interest

All authors declare that they have no known competing financial interests or personal relationships that could have appeared to influence the work reported in this paper.
