# Supplementary material for: A secondary analysis investigating therapist-effects in a guided internet-based intervention for adults with symptoms of depression
Source: Internet Interv. 2026 Jun 16;45:100964. doi: 10.1016/j.invent.2026.100964 (PMC13284498; doi:10.1016/j.invent.2026.100964)
Supplement: Supplementary file 1 — Supplementary material [file mmc1.pdf]

## Supplemental Material 1 – Baseline Variables per IBI Therapist

The distribution of baseline PHQ-9 depression severity and age per IBI therapist.

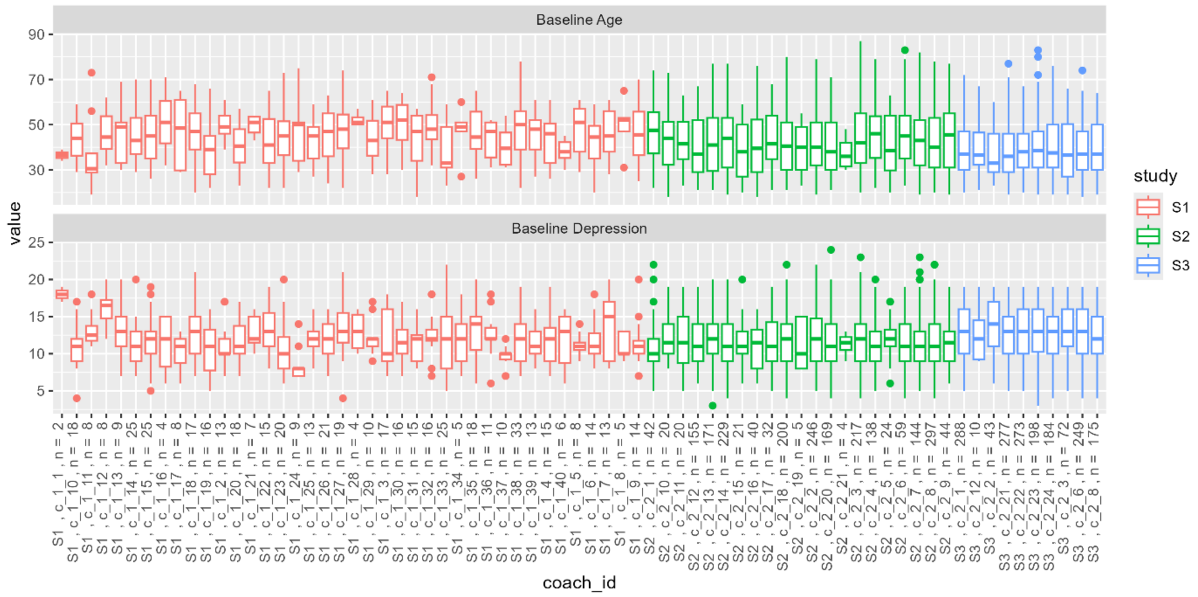


The rate of females and individuals with a diagnosis of MDE per IBI therapist.

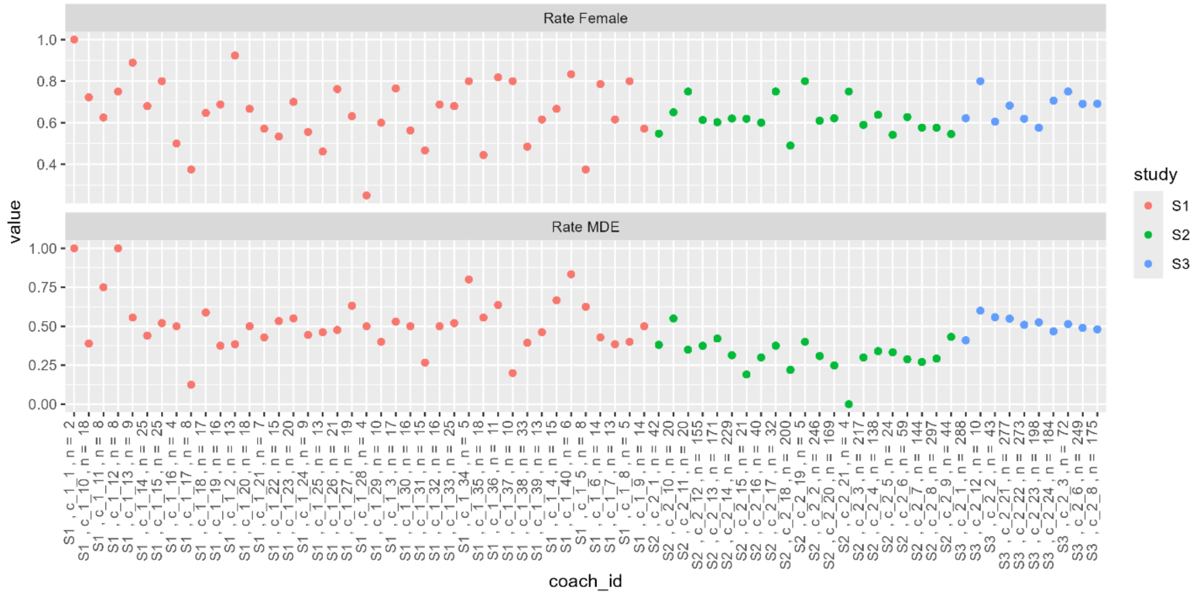

**Supplemental Material 2 – Model Equations**

Heterogeneity in continuous outcome variables was estimated using the following multilevel model:

$$y_{ij} = \beta_0 + \boldsymbol{x}_i^T \boldsymbol{\beta} + U_j + e_{ij}$$

with $y_{ij}$ the outcome for individual $i$ treated by therapist $j$, $\boldsymbol{x}_i$ is a vector of person-specific covariate values including the age, sex, and baseline depression score for individual $i$, $\boldsymbol{\beta}$, a vector of corresponding fixed effect regression coefficients, $U_j$ a therapist-specific random effect, and $e_{ij}$ an error term. The model assumes $U_j \sim N(0, \tau_0^2)$ and $e_{ij} \sim N(0, \tau_e^2)$.

For binary outcome variables, a logistic multilevel model was used:

$$log\left(\frac{p_{ij}}{1 - p_{ij}}\right) = \beta_0 + \boldsymbol{x}_i^T \boldsymbol{\beta} + U_j$$

with the left side being the log-odds for individual $i$ treated by therapist $j$.

To estimate the association between the number of treated clients and the feedback length with the outcomes, the following model was used:

$$y_{ij} = \beta_0 + \boldsymbol{x}_i^T \boldsymbol{\beta} + x_{ij}^{NT} \beta^{NT} + \beta_W^{FL}\left(x_{ij}^{FL} - \bar{x}_j{}^{FL}\right) + \beta_B^{FL} \bar{x}_j{}^{FL} + U_j + e_{ij}$$

With $x_{ij}^{NT}$ the within-therapist rank of individual $i$ within the sequence of patients treated by therapist $j$ and $\beta^{NT}$ being the corresponding fixed-effects regression weight. While $\bar{x}_j{}^{FL}$ represents the therapist-specific average feedback length, $\left(x_{ij}^{FL} - \bar{x}_j{}^{FL}\right)$ represents the deviation of the length of a given feedback from the therapist's average feedback length. $\beta_W^{FL}$ and $\beta_B^{FL}$ are the corresponding regression weights. For simplicity, the equation presents the predictors FL and NT in a single model, although the models were estimated separately for each predictor. The logistic multilevel models follow the same structure.

**Supplemental Material 3 – Derivation of the Median Absolute Difference (MAD)**

The MAD follows the logic presented for deriving the MOR. It exploits the assumption that the random effects are normally distributed. Consider a multilevel model $Y_{ij} = \boldsymbol{x}_i^T\boldsymbol{\beta} + U_j + e_{ij}$, with $Y_{ij}$ the outcome for individual $i$ in therapist $j$, $\boldsymbol{x}_i^T$ a vector of person-specific covariate values, $\boldsymbol{\beta}$, a vector of fixed-effects regression coefficients, $U_j$ a random effect, and $e_{ij}$ an error term. Assume $U_j \sim N(0, \tau_0^2)$ and $e_{ij} \sim N(0, \tau_e^2)$. The expected outcome for an individual $i$ treated by therapist $j$ is $E(Y_{ij}|U_j = u_j) = \boldsymbol{x}_i^T\boldsymbol{\beta} + u_j$. If two therapists $j$ and $k$ could treat the same client, the difference in the expected values equals the difference in the random effects: $E(Y_{ij}|U_j = u_j) - E(Y_{ik}|U_k = u_k) = (\boldsymbol{x}_i^T\boldsymbol{\beta} + u_j) - (\boldsymbol{x}_i^T\boldsymbol{\beta} + u_k) = u_j - u_k$. Given that $U_j$ and $U_k$ are independent and normally distributed, $U_j - U_k \sim N(0, 2\tau_0^2)$ and $|U_j - U_k|$ follows a folded normal distribution. Standardizing $U_j - U_k$ results in a standard normally distributed variable $Z = (U_j - U_k)/\sqrt{(2\tau_0^2)} \sim N(0,1)$. We are interested in $p = P(-z \leq Z \leq z)$. That is, the probability that $Z$ is between $-z$ and $z$. This gives $p = P(-z \leq Z \leq z) = P(Z \leq z) - [1 - P(Z \leq z)] = 2 \times P(Z \leq z) - 1$, which implies $z = \phi^{-1}\left(\frac{p+1}{2}\right)$ with $\phi^{-1}$ being the quantile function of the standard normal distribution. With *p* = 0.5, $(-z, z)$ covers the central 50% of the standard normal distribution. If the symmetric distribution is folded at zero, the interval $[0, z)$ must comprise 50% of the folded distribution. Thus, $z$ is the median of the folded standard normal distribution. It is necessary to transform $z$ back to the original scale metric to receive the $MAD = \sqrt{2} \times \tau_0 \times \phi^{-1}\left(\frac{0.5+1}{2}\right)$. Note that the MAD does not depend on $\tau_e^2$. Two trials with the same random-effects variance and the same outcome measure will have the same MAD, even if $\tau_e^2$ differs. This is not true of the $ICC = \tau_0^2/(\tau_0^2 + \tau_e^2)$ which depends on $\tau_e^2$.

# Supplemental Material 4 - Tables

**Supplemental Table 4.1**

Association of Treatment Discontinuation, Response, and Change with the Number of Treated Clients in RCT2 and RCT3

| Criterion | Parameter | RCT1<br>β [95% CRI] | RCT2<br>β [95% CRI] | RCT3<br>β [95% CRI] |
|---|---|---|---|---|
| Dropout | Intercept | −2.08 [−2.54, −1.66] | −1.32 [−1.50, −1.13] | −1.35 [−1.56, −1.14] |
| | Female (vs. Male) | 0.52 [0.01, 1.02] | 0.17 [−0.04, 0.37] | 0.30 [0.08, 0.52] |
| | (Age – 40) | −0.01 [−0.03, 0.01] | −0.02 [−0.02, −0.01] | −0.02 [−0.03, −0.01] |
| | (PHQ−9 – 10) | 0.07 [0.00, 0.14] | 0.01 [−0.02, 0.04] | 0.02 [−0.01, 0.05] |
| | $\beta_{CRF}$ | --- | −0.06 [−0.26, 0.14] | --- |
| | $\beta_{NT}$ | --- | --- | 0.29 [0.07, 0.51] |
| | $\tau_0$ | 0.48 [0.05, 0.93] | 0.08 [0.00, 0.24] | 0.09 [0.00, 0.27] |
| Meaningful Improvement | Intercept | −0.44 [−0.72, −0.17] | −0.55 [−0.71, −0.39] | −0.60 [−0.79, −0.41] |
| | Female (vs. Male) | −0.12 [−0.50, 0.25] | −0.09 [−0.27, 0.08] | −0.26 [−0.48, −0.03] |
| | (Age – 40) | 0.00 [−0.02, 0.02] | 0.01 [0.00, 0.01] | 0.01 [−0.00, 0.01] |
| | (PHQ−9 – 10) | −0.01 [−0.07, 0.04] | −0.01 [−0.04, 0.01] | −0.03 [−0.06, −0.01] |
| | $\beta_{CRF}$ | --- | −0.07 [−0.25, 0.10] | --- |
| | $\beta_{CAU}$ | --- | --- | −0.30 [−0.52, −0.08] |
| | $\tau_0$ | 0.29 [0.02, 0.63] | 0.09 [0.00, 0.26] | 0.09 [0.00, 0.27] |
| Change | Intercept | −3.44 [−3.98, −2.90] | −3.45 [−3.80, −3.12] | −3.20 [−3.55, −2.84] |
| | Female (vs. Male) | 0.20 [−0.58, 0.98] | 0.29 [−0.06, 0.63] | 0.25 [−0.15, 0.65] |
| | (Age – 40) | −0.00 [−0.03, 0.03] | −0.01 [−0.03, −0.00] | −0.01 [−0.03, 0.01] |
| | (PHQ−9 – 10) | −0.26 [−0.37, −0.16] | −0.15 [−0.20, −0.10] | −0.19 [−0.24, −0.14] |
| | $\beta_{CRF}$ | --- | 0.31 [−0.04, 0.65] | --- |
| | $\beta_{NT}$ | --- | --- | 0.76 [0.37, 1.15] |
| | $\tau_0$ | 0.29 [0.01, 0.79] | 0.24 [0.02, 0.55] | 0.13 [0.01, 0.41] |
| | $\sigma^2$ | 4.31 [4.06, 4.57] | 4.16 [4.04, 4.28] | 4.12 [3.99, 4.26] |

*Note.* β = Median of the parameters' posterior distribution. OR = Odds ratio. 95% CI = 95% Credibility interval.

**Supplemental Table 4.2**

The Association of Treatment Discontinuation, Response, and Change with the Number of Treated Clients in RCT2 and RCT3

| Trial | Criterion | Parameter | β [95% CRI] | OR [95% CRI] |
|---|---|---|---|---|
| RCT2 | Dropout | Intercept | −1.367 [−1.595, −1.145] | |
| | | (Age – 40) | −0.015 [−0.023, −0.007] | 0.985 [0.977, 0.993] |
| | | (PHQ−9 – 10) | 0.011 [−0.018, 0.040] | 1.011 [0.982, 1.041] |
| | | Female (vs. Male) | 0.163 [−0.038, 0.367] | 1.184 [0.962, 1.443] |
| | | $\beta_{CRF}$ | −0.061 [−0.261, 0.134] | 0.945 [0.770, 1.143] |
| | | $\beta_{NT}$ | 0.010 [−0.016, 0.035] | 1.010 [0.984, 1.036] |
| | | $\tau_0$ | 0.079 [0.003, 0.246] | |
| RCT3 | Dropout | Intercept | −1.489 (−1.759, −1.226) | |
| | | (Age – 40) | −0.021 (−0.031, −0.011) | 0.980 (0.970, 0.989) |
| | | PHQ−9 | 0.022 (−0.005, 0.049) | 1.022 (0.995, 1.050) |
| | | Female (vs. Male) | 0.299 (0.081, 0.525) | 1.358 (1.084, 1.690) |
| | | $\beta_{CRF}$ | 0.263 (0.038, 0.488) | 1.309 (1.039, 1.629) |
| | | $\beta_{NT}$ | 0.023 (−0.004, 0.049) | 1.023 (0.996, 1.051) |
| | | $\tau_0$ | 0.092 (0.004, 0.285) | |
| RCT2 | Meaningful Improvement | Intercept | −0.476 [−0.672, −0.283] | |
| | | (Age – 40) | 0.006 [−0.000, 0.013] | 1.006 [1.000, 1.013] |
| | | (PHQ−9 – 10) | −0.012 [−0.039, 0.013] | 0.988 [0.962, 1.013] |
| | | Female (vs. Male) | −0.089 [−0.266, 0.087] | 0.918 [0.767, 1.091] |
| | | $\beta_{CRF}$ | −0.072 [−0.244, 0.097] | 0.934 [0.783, 1.102] |
| | | $\beta_{NT}$ | −0.015 [−0.039, 0.007] | 0.985 [0.962, 1.007] |
| | | $\tau_0$ | 0.097 [0.004, 0.269] | |
| RCT3 | Meaningful Improvement | Intercept | −0.599 [−0.844, −0.353] | |
| | | (Age – 40) | 0.006 [−0.003, 0.014] | 1.006 [0.997, 1.015] |
| | | (PHQ−9 – 10) | −0.032 [−0.059, −0.005] | 0.968 [0.943, 0.994] |
| | | Female (vs. Male) | −0.256 [−0.480, −0.036] | 0.779 [0.619, 0.965] |
| | | $\beta_{CRF}$ | −0.301 [−0.517, −0.091] | 0.744 [0.597, 0.913] |
| | | $\beta_{NT}$ | −0.000 [−0.025, 0.025] | 1.000 [0.975, 1.025] |
| | | $\tau_0$ | 0.086 [0.003, 0.268] | |
| RCT2 | Change | Intercept | −3.733 [−4.118, −3.334] | |
| | | (Age – 40) | −0.013 [−0.026, 0.001] | |
| | | (PHQ−9 – 10) | −0.150 [−0.202, −0.099] | |
| | | Female (vs. Male) | 0.272 [−0.072, 0.617] | |
| | | $\beta_{CRF}$ | 0.301 [−0.043, 0.639] | |
| | | $\beta_{NT}$ | 0.058 [0.013, 0.103] | |
| | | $\tau_0$ | 0.241 [0.018, 0.549] | |
| | | $\varepsilon_0$ | 4.154 [4.037, 4.277] | |
| RCT3 | Change | Intercept | −3.170 [−3.624, −2.718] | |
| | | (Age – 40) | −0.010 [−0.027, 0.006] | |
| | | (PHQ−9 – 10) | −0.192 [−0.242, −0.144] | |
| | | Female (vs. Male) | 0.255 [−0.156, 0.666] | |
| | | $\beta_{CRF}$ | 0.771 [0.365, 1.171] | |
| | | $\beta_{NT}$ | −0.005 [−0.052, 0.043] | |
| | | $\tau_0$ | 0.132 [0.005, 0.429] | |
| | | $\sigma^2$ | 4.122 [3.991, 4.258] | |

*Note.* β = Median of the parameters' posterior distribution. OR = Odds ratio. 95% CRI = 95% Credibility interval.

**Supplemental Table 4.3**

The Association of Treatment Discontinuation, Response, and Change with Individualization in RCT2 and 3.

| Trial | Criterion | Parameter | β [95% CI] | OR [95% CI] |
|---|---|---|---|---|
| RCT1 | Dropout | Intercept | 1.796 [−0.516, 4.142] | |
| | | (Age – 40) | −0.010 [−0.038, 0.017] | 0.990 [0.963, 1.018] |
| | | (PHQ−9 – 10) | 0.094 [0.005, 0.186] | 1.100 [1.005, 1.205] |
| | | Female (vs. Male) | 0.526 [−0.108, 1.158] | 1.783 [0.897, 3.185] |
| | | $IND_W$ | −0.074 [−0.487, 0.319] | 0.948 [0.614, 1.376] |
| | | $IND_B$ | −0.879 [−1.358, −0.431] | 0.427 [0.257, 0.650] |
| | | $\tau_0$ | 0.399 [0.019, 0.974] | |
| RCT3 | Dropout | Intercept | −1.631 [−2.685, −0.504] | |
| | | (Age – 40) | −0.023 [−0.034, −0.011] | 0.978 [ 0.966, 0.989] |
| | | (PHQ−9 – 10) | 0.039 [0.007, 0.072] | 1.040 [1.007, 1.074] |
| | | Female (vs. Male) | 0.207 [−0.065, 0.479] | 1.242 [0.937, 1.615] |
| | | PAF (vs. WL) | 0.051 [−0.220, 0.323] | 1.063 [0.802, 1.381] |
| | | $IND_W$ | −0.258 [−0.418, −0.101] | 0.775 [0.658, 0.904] |
| | | $IND_B$ | −0.024 [−0.239, 0.171] | 0.982 [0.787, 1.186] |
| | | $\tau_0$ | 0.174 [0.008, 0.484] | |
| RCT1 | Response | Intercept | −0.837 [−2.156, 0.458] | |
| | | (Age – 40) | −0.002 [−0.018, 0.015] | 0.998 [0.982, 1.015] |
| | | (PHQ−9 – 10) | −0.011 [−0.066, 0.044] | 0.989 [0.936, 1.045] |
| | | Female (vs. Male) | −0.089 [−0.479, 0.296] | 0.933 [0.619, 1.345] |
| | | $IND_W$ | −0.010 [−0.216, 0.196] | 0.995 [0.805, 1.216] |
| | | $IND_B$ | 0.092 [−0.146, 0.336] | 1.105 [0.864, 1.399] |
| | | $\tau_0$ | 0.303 [0.021, 0.659] | |
| RCT3 | Response | Intercept | −0.312 (−1.060, 0.500) | |
| | | (Age – 40) | 0.004 (−0.005, 0.013) | 1.004 (0.995, 1.013) |
| | | (PHQ−9 – 10) | −0.036 (−0.064, −0.009) | 0.964 (0.938, 0.991) |
| | | Female (vs. Male) | −0.209 (−0.433, 0.018) | 0.817 (0.649, 1.018) |
| | | PAF (vs. WL) | −0.221 (−0.442, 0.002) | 0.807 (0.642, 1.002) |
| | | $IND_W$ | 0.070 (−0.055, 0.195) | 1.075 (0.946, 1.215) |
| | | $IND_B$ | −0.035 (−0.184, 0.103) | 0.968 (0.832, 1.108) |
| | | $\tau_0$ | 0.113 (0.004, 0.336) | |
| RCT1 | Change | Intercept | −3.197 [−5.589, −0.782] | |
| | | (Age – 40) | 0.001 [−0.031, 0.034] | |
| | | (PHQ−9 – 10) | −0.281 [−0.396, −0.166] | |
| | | Female (vs. Male) | 0.139 [−0.646, 0.929] | |
| | | $IND_W$ | −0.099 [−0.538, 0.336] | |
| | | $IND_B$ | −0.084 [−0.518, 0.347] | |
| | | $\tau_0$ | 0.296 [0.012, 0.813] | |
| | | $\sigma$ | 4.325 [4.067, 4.608] | |
| RCT3 | Change | Intercept | −3.523 [−4.911, −2.068] | |
| | | (Age – 40) | −0.008 [−0.026, 0.010] | |
| | | (PHQ−9 – 10) | −0.208 [−0.261, −0.155] | |
| | | Female (vs. Male) | 0.130 [−0.309, 0.569] | |
| | | PAF (vs. WL) | 0.622 [0.192, 1.054] | |
| | | $IND_W$ | −0.213 [−0.456, 0.026] | |
| | | $IND_B$ | 0.027 [−0.240, 0.287] | |
| | | $\tau_0$ | 0.179 [0.006, 0.596] | |
| | | $\sigma$ | 4.210 [4.068, 4.358] | |

*Note.* β = Median of the parameters' posterior distribution. OR = Odds ratio. 95% CI = 95% Credibility interval. W = within-therapist centered amount feedback length. B = average feedback length per therapist at the between−level.

## Supplemental Material 5

We conducted various additional analyses. The following models were estimated:

- Model 1 corresponds to the model reported in the Manuscript. It includes age (centered at 40 years), sex, and baseline symptom severity cetnered at 10 as covariates, along with the intervention arm for RCT2 and RCT3. If it was run on the pooled dataset, it also included dummy-coded "study" as a covariate (Reference: RCT1).
- Model 2 extends Model 1 by additionally adjusting for calendar time (year quarters) and the overall number of treated clients by a given therapist. Both covariates could be considered for RCT1, RCT2, and RCT3, but not in the pooled dataset. Therefore, the results reported under 'combined' include only the overall number of treated clients as an additional covariate.
- Model 3 replicates Model 1 but is estimated separately within each treatment arm. Note that this ignores that the therapists treated clients from both treatment arms in parallel.
- Model 4 includes only individuals who started to work with all treatment modules.

All parameter estimates are reported in the OSF.

**Supplemental Table 5.1**

Sensitivity Analyses

| **Criterion** | | **RCT1**<br>β [95% CRI] | **RCT2**<br>β [95% CRI] | **RCT3**<br>β [95% CRI] | **Combined**<br>β [95% CRI] |
|---|---|---|---|---|---|
| ***Discontinuation*** | | | | | |
| Model 1 | cVPC | 0.075 [0.001, 0.209] | 0.003 [0.000, 0.017] | 0.004 [0.000, 0.021] | 0.002 [0.000, 0.009] |
| Model 2 | cVPC | 0.101 [0.003, 0.258] | 0.004 [0.000, 0.022] | 0.005 [0.000, 0.031] | 0.002 [0.000, 0.009] |
| Model 3 – Arm 1 | cVPC | 0.075 [0.001, 0.209] | 0.033 [0.001, 0.110] | 0.008 [0.000, 0.045] | N/A |
| Model 3 – Arm 2 | cVPC | N/A | 0.008 [0.000, 0.040] | 0.009 [0.000, 0.053] | N/A |
| Model 1 | cMOR | 1.617 [1.045, 2.436] | 1.081 [1.002, 1.257] | 1.088 [1.003, 1.288] | 1.060 [1.002, 1.181] |
| Model 2 | cMOR | 1.783 [1.104, 2.774] | 1.088 [1.003, 1.299] | 1.107 [1.004, 1.360] | 1.058 [1.002, 1.178] |
| Model 3 – Arm 1 | cMOR | 1.617 [1.045, 2.436] | 1.354 [1.043, 1.839] | 1.136 [1.005, 1.458] | N/A |
| Model 3 – Arm 2 | cMOR | N/A | 1.134 [1.005, 1.424] | 1.149 [1.006, 1.504] | N/A |
| ***Response*** | | | | | |
| Model 1 | cVPC | 0.031 [0.000, 0.108] | 0.004 [0.000, 0.019] | 0.004 [0.000, 0.021] | 0.002 [0.000, 0.010] |
| Model 2 | cVPC | 0.036 [0.000, 0.118] | 0.006 [0.000, 0.030] | 0.005 [0.000, 0.031] | 0.002 [0.000, 0.011] |
| Model 3 – Arm 1 | cVPC | 0.031 [0.000, 0.108] | 0.005 [0.000, 0.024] | 0.007 [0.000, 0.036] | N/A |
| Model 3 – Arm 2 | cVPC | N/A | 0.007 [0.000, 0.036] | 0.009 [0.000, 0.054] | N/A |
| Model 4 | cVPC | 0.030 [0.000, 0.111] | 0.006 [0.000, 0.027] | 0.004 [0.000, 0.021] | 0.003 [0.000, 0.014] |
| Model 1 | cMOR | 1.332 [1.017, 1.824] | 1.095 [1.004, 1.276] | 1.088 [1.003, 1.289] | 1.166 [1.003, 1.188] |
| Model 2 | cMOR | 1.366 [1.020, 1.882] | 1.118 [1.005, 1.354] | 1.107 [1.004, 1.360] | 1.069 [1.003, 1.196] |
| Model 3 – Arm 1 | cMOR | 1.332 [1.017, 1.824] | 1.100 [1.003, 1.310] | 1.120 [1.004, 1.396] | N/A |
| Model 3 – Arm 2 | cMOR | N/A | 1.125 [1.004, 1.397] | 1.143 [1.005, 1.512] | N/A |
| Model 4 | cMOR | 1.322 [1.015, 1.814] | 1.114 [1.005, 1.333] | 1.088 [1.003, 1.289] | 1.083 [1.004, 1.230] |
| ***Change*** | | | | | |
| Model 1 | cICC | 0.007 [0.000, 0.033] | 0.005 [0.000, 0.017] | 0.002 [0.000, 0.010] | 0.001 [0.000, 0.006] |
| Model 2 | cICC | 0.007 [0.000, 0.036] | 0.006 [0.000, 0.021] | 0.002 [0.000, 0.012] | 0.001 [0.000, 0.006] |
| Model 3 – Arm 1 | cICC | 0.007 [0.000, 0.033] | 0.010 [0.000, 0.037] | 0.003 [0.000, 0.019] | N/A |
| Model 3 – Arm 2 | cICC | N/A | 0.003 [0.000, 0.014] | 0.004 [0.000, 0.024] | N/A |
| Model 4 | cICC | 0.012 [0.000, 0.056] | 0.003 [0.000, 0.015] | 0.003 [0.000, 0.016] | 0.001 [0.000, 0.006] |
| Model 1 | cMAD | 0.275 [0.010, 0.753] | 0.234 [0.018, 0.526] | 0.121 [0.004, 0.393] | 0.124 [0.006, 0.306] |
| Model 2 | cMAD | 0.287 [0.012, 0.783] | 0.267 [0.024, 0.582] | 0.130 [0.004, 0.428] | 0.128 [0.006, 0.319] |
| Model 3 – Arm 1 | cMAD | 0.275 [0.010, 0.753] | 0.335 [0.021, 0.769] | 0.179 [0.007, 0.579] | N/A |
| Model 3 – Arm 2 | cMAD | N/A | 0.173 [0.007, 0.475] | 0.187 [0.007, 0.577] | N/A |
| Model 4 | cMAD | 0.344 [0.015, 0.932] | 0.181 [0.008, 0.500] | 0.178 [0.006, 0.545] | 0.114 [0.005, 0.315] |

## Supplemental Material 6

For discontinuation, two further outcomes were explored.

- Model 1 corresponds to the results reported in the Manuscript, with "having started to work with all treatment modules" as the outcome. All models included baseline symptom severity, age, and sex as covariates, as well as intervention arm for RCT2 and RCT3. When analyses were conducted on the pooled dataset, a dummy-coded study variable was also included as a covariate (reference: RCT1).
- Model 2 treated the number of completed modules as a continuous outcome variable.
- Model 3 considered discontinuation after the first therapist contact as the outcome. Given that modules were completed sequentially, this is equivalent to discontinuing the program before gaining access to the second treatment module. Because the number of events was small in RCT1, this analysis was conducted only for RCT2, RCT3, and the pooled sample of RCT2 and RCT3.

**Supplemental Table 6.1**

Sensitivity Analysis for Discontinuation

| Criterion | | RCT1<br>β [95% CRI] | RCT2<br>β [95% CRI] | RCT3<br>β [95% CRI] | Combined<br>β [95% CRI] |
|---|---|---|---|---|---|
| Discontinuation | | | | | |
| Model 1 | cVPC | 0.075 [0.001, 0.209] | 0.003 [0.000, 0.017] | 0.004 [0.000, 0.021] | 0.002 [0.000, 0.009] |
| Model 2 | cICC | 0.023 [0.000, 0.076] | 0.001 [0.000, 0.008] | 0.002 [0.000, 0.010] | 0.001 [0.000, 0.004] |
| Model 3 | cVPC | N/A | 0.017 [0.000, 0.085] | 0.011 [0.000, 0.064] | 0.006 [0.000, 0.032] |
| Model 1 | cMOR | 1.617 [1.045, 2.436] | 1.081 [1.002, 1.257] | 1.088 [1.003, 1.288] | 1.060 [1.002, 1.181] |
| Model 2 | cMAD | 0.182 [0.013, 0.384] | 0.045 [0.002, 0.135] | 0.051 [0.002, 0.165] | 0.031 [0.001, 0.093] |
| Model 3 | cMOR | N/A | 1.214 [1.007, 1.696] | 1.163 [1.005, 1.573] | 1.120 [1.004, 1.370] |
